# Human papillomavirus vaccine knowledge and beliefs among school health staff and principals in Cape Town, South Africa

**DOI:** 10.4102/hsag.v30i0.3144

**Published:** 2025-12-02

**Authors:** Maria Jose, Dean B. João, Amira Botha, Mihle Gadu, Zoë E. Mousley

**Affiliations:** 1 School of Public Health, Faculty of Health Sciences, University of Cape Town, Cape Town, South Africa

**Keywords:** human papillomavirus, vaccination, school health, health education, knowledge, beliefs, cervical cancer, South Africa

## Abstract

**Background:**

South Africa has implemented school-based human papillomavirus (HPV) vaccination for female-bodied learners at public schools since 2014. However, vaccine uptake has declined in recent years.

**Aim:**

To describe the knowledge and beliefs about HPV and HPV vaccination held by school health representatives (SHRs) and principals at schools hosting HPV vaccine campaigns Mitchells Plain, South Africa.

**Setting:**

Schools in Mitchells Plain, South Africa, that have hosted at least one HPV vaccination campaign since 2021.

**Methods:**

A cross-sectional study was conducted using a questionnaire administered to SHRs and principals from 48 schools selected through convenience sampling.

**Results:**

Of 55 responses (52.1% of eligible schools), 76.4% of participants were teachers, 16.7% principals and 9.1% administrators. Majority (85.5%) would recommend HPV vaccination to students. However, knowledge scores were low with a median score of 7 (interquartile range [IQR] = 4) out of 19. Administrative staff scored the highest. Although participants had participated in an average of 3.55 vaccination campaigns, only 20% reported receiving HPV-related training. The majority (91.9%) felt ill-equipped to counsel others about HPV vaccination. No relationship was found between the number of vaccine campaigns participated in and knowledge scores.

**Conclusion:**

Although supportive of HPV vaccine campaigns, SHRs and principal staff lack knowledge on HPV and HPV vaccination programmes.

**Contribution:**

This research identifies critical knowledge gaps and emphasises the need for targeted educational approaches to enhance public health education regarding HPV and HPV vaccination.

## Introduction

In 2017, cervical cancer was reported as being the fourth most frequently diagnosed cancer, the fourth leading cause of cancer deaths in women globally and the leading cause of cancer-related deaths in South Africa (Amponsah-Dacosta et al. 2022; Zhao et al. 2021). Although the absolute number of cervical cancer cases, disability-adjusted life years and deaths rose between 2007 and 2017, the age-standardised rates for incidence, disability-adjusted life years and deaths dropped (Zhao et al. 2021). This is speculated to be because of increased provision of human papillomavirus (HPV) vaccination, among other factors (Zhao et al. 2021). There is a notable difference in the incidence of HPV infection and cervical cancer between high-income countries with good HPV vaccination coverage and low- and middle-income countries with poorer access to healthcare (Zhao et al. 2021).

In South Africa, cervical cancer is the most common cancer in women aged 15–44 years (Bruni et al. 2023). Almost all cases of cervical cancer are caused by HPV. Although most cases of infection are cleared by the body’s immune system, around 2% of infections with oncogenic strains of HPV persist in the cervix and, in the presence of additional risk factors such as HIV co-infection, can lead to premalignant and malignant lesions of the cervix (Olorunfemi et al. 2018).

In response to the high burden of cervical cancer in South Africa, the Department of Health provides three cervical smear screenings in a woman’s lifetime, provided once every 10 years, to all women over the age of 30 using public healthcare services. The current HPV vaccination campaign, whereby all female-bodied learners aged 9 years–12 years attending public schools are provided with the opportunity to receive the HPV vaccine free of charge, was introduced in 2014 and is delivered jointly by the Expanded Programme on Immunisation and the Integrated School Health Programme (South African National Department of Health 2017). The HPV vaccine currently used for this programme covers HPV-16 and HPV-18 (which are responsible for over 70% of cervical cancer cases) with 98.2% efficacy (South African National Department of Health 2017).

Originally given as two doses 6 months apart, it is given as a single dose as of 2024. At the inception of the programme, in 2014, the first dose was well received with an uptake of 86.6% nationally, although the second dose had a lower coverage of 65% (Khosa et al. 2022; Statistics South Africa 2023). The momentum of the programme has come under scrutiny in recent years as by 2021, national coverage was just 37% and 34% for the first and second doses, respectively, a trend mirrored in the Western Cape (Western Cape Department of Health and Wellness 2016, 2023). This poor coverage was attributed to a range of challenges including over-extension of services responding to concurrent measles outbreaks, as well as fewer consent forms being returned and hesitancy by caregivers to sign consent (Western Cape Department of Health and Wellness 2023).

Consent forms have, in fact, emerged as a critical administrative factor influencing HPV vaccination uptake. Studies in South Africa have identified low return rates of consent forms and caregiver reluctance to sign them as key barriers to successful vaccination delivery (Western Cape Department of Health and Wellness 2023).

International research similarly highlights the significant role of those responsible for managing consent forms, with evidence from the United Kingdom finding that those responsible for consent forms had an enormous impact on the success of the campaign (Brabin et al. 2011). In a study conducted in Canada, school nurses and managers noted that teachers could positively impact vaccination uptake by ensuring that consent forms were sent home and returned (Dubé et al. 2019). Other potential barriers to vaccine uptake included vaccine hesitancy (often fuelled by misinformation on social media), a concern amplified in the post-coronavirus disease 2019 (COVID-19) era, where vaccine hesitancy has gained global attention, a lack of accurate information regarding cervical cancer and its link to HPV infection, fear that vaccination may increase early or unsafe sexual behaviour and high cost of vaccines (Amponsah-Dacosta et al. 2022; Dorji et al. 2021; Hossain et al. 2021; Jeudin et al. 2014; Khosa et al. 2022; Newman et al. 2018; Ngcobo et al. 2018).

Globally, the factors that increased vaccine uptake in both low- and middle-income countries and high-income countries included belief in the benefits of HPV vaccination, clear and effective education, recommendation by a trusted healthcare provider, free access to vaccines and strategies to provide catch-up vaccinations to those who had missed the opportunity initially (Amponsah-Dacosta et al. 2022; Gallagher et al. 2017; Jeudin et al. 2014; Ngcobo et al. 2018). The factors influencing vaccine uptake globally were largely similar to those found impacting uptake in South Africa, where education, catch-up programmes and free and convenient access to HPV vaccination were associated with increased uptake (Amponsah-Dacosta et al. 2022; Dubé et al. 2019; Katz et al. 2013; Lenkokile, Hlongwane & Clapper 2019; Ngcobo et al. 2018).

For this reason, teachers, school principals and school administration staff have a direct impact on the delivery of vaccinations to children. School principals have been noted anecdotally to have an impact on vaccination uptake, with school nurses noticing a higher uptake in schools where the principal ‘dynamically promoted’ the vaccine (Brabin et al. 2011). The degree to which teachers educate their students and address students’ anxieties regarding vaccinations is also a determining factor in vaccination uptake (Dubé et al. 2019). Many HPV vaccination campaigns around the world, including the one in South Africa, are based in schools.

To enable the success of school-based vaccination campaigns, teachers and principals must have a high degree of knowledge regarding HPV, HPV vaccination and cervical cancer. Conversely, a lack of knowledge is likely to negatively impact vaccination rates. Empowering these key role-players via educational interventions may be a useful strategy to increase uptake.

However, literature investigating the knowledge, attitudes and practices of teachers and school principals with respect to HPV vaccination campaigns in South Africa is lacking. This study addresses this gap by exploring these perspectives to inform future strategies for school-based vaccination campaigns.

## Research aim

The study aimed to describe the knowledge and beliefs about HPV and HPV vaccination among school principals and school health representatives (SHRs) at schools that currently host HPV vaccine campaigns in Mitchells Plain, South Africa.

### Study objectives

- To describe the knowledge and beliefs about HPV and HPV vaccination held by school principals and SHRs working at public schools.
- To identify potential barriers in the promotion of HPV vaccine uptake among SHRs and principal staff.
- To demonstrate heterogeneity in HPV-related knowledge and beliefs of SHRs and principal staff.

## Research methods and design

### Study design

This research employed a cross-sectional quantitative study design. This design was selected for its efficiency, cost-effectiveness and ability to assess exposure and outcomes simultaneously while noting its inability to establish temporality between exposures and outcomes (Mann 2003; Etikan, Musa & Alkassim 2016).

### Setting

The research was conducted in Mitchells Plain, Western Cape province of South Africa. In 2020, the Western Cape Government estimated the population of the area to be over 615 000, with a large portion in the working-age group (15–64 years old) (Statistics South Africa 2011; Western Cape Department of Health and Wellness 2020). Much of the population speaks Afrikaans, with English being the second most common language. In the area, there are 48 public primary schools, and the study participants were selected from these schools.

### Population and sampling methods

The study targeted teachers and administrative staff appointed as SHRs at their place of work, who serve as liaisons between the schools and the nurses running the campaigns. Among the identified schools, one to two SHR staff are appointed per school. Principals were also identified for recruitment from each school. An estimated sample size of 98 eligible participants was identified for study recruitment (i.e. two persons from each school, one SHR and one principal). Eligible participants had to be actively employed at their respective schools between 2021 and 2024 and had to have participated in at least one round of the National School-Based HPV vaccination campaign, which included roles such as organising vaccination sessions, coordinating with healthcare professionals, obtaining parental consent and supporting students and parents. A convenience sampling strategy was used, and all 48 public primary schools in the area that had hosted the HPV campaign since 2021 were invited to participate in the study. All schools, regardless of language of instruction, were eligible to participate in this study. The Western Cape Education Department and the Metro South Education District Office were contacted and provided with the study ethics approval contract via email for study approval. Thereafter, all primary schools in Mitchells Plain that hosted HPV vaccine campaigns were contacted. Principals at each selected school were informed of the study and provided with the study’s research approval contract via email, and an in-person information session was hosted to introduce the study in which approximately 65% of recruited schools had a representative in attendance. A similar participant recruitment launch event was hosted for SHRs, which was attended by approximately 70% of recruited schools.

### Data collection methods and processes

The study utilised a structured questionnaire adapted from validated tools used in previous studies on HPV vaccine knowledge, awareness and vaccination attitudes among educational staff (Kim 2012; Masika et al. 2015; Whelan et al. 2014). The questionnaire is available in supplementary materials, Appendix 1. Questions were selected and modified to meet study objectives and reflect the South African school context. Key demographic variables were included, with consolidated questions addressing HPV vaccination for participants and their children to avoid redundancy. Questions assessing school type, fee structure, health-promoting school status and school gender composition were also included.

Human papillomavirus knowledge was assessed by 19 items of an HPV knowledge scale, the construct validity of which was confirmed among Korean health teachers (Kim 2012). Questions in this section remained consistent with original validated tools, with minor language adjustments for clarity, omission of duplicate questions and the addition of a question on the cervical smear schedule available at public health facilities in South Africa. Each question followed a single-best-answer format with response options of ‘True’, ‘False’ or ‘Do not know’. Correct answers were scored as one point, while incorrect or ‘Do not know’ responses were scored as zero. A continuous variable, ‘Knowledge Uncertainty’, was constructed for analysis as an integer count of the number of ‘Do not know’ responses submitted across the 19-question knowledge section.

Health beliefs towards HPV vaccination were assessed using seven validated statements from the source study, with response options of ‘agree’, ‘disagree’ or ‘neutral’ (Masika et al. 2015). Health beliefs in this study were adapted to fit the present study sample. Participants were asked about their intention to recommend the vaccine, with response options of ‘Yes’, ‘I am not sure’ or ‘No’. Those who selected ‘No’ were prompted to qualify their vaccine hesitant views by selecting from five predefined statements. New variables, related to roles and involvement in vaccination campaigns, were introduced to align with participants’ responsibilities within the health-promoting school setting. Additionally, a non-scoring question assessed participants’ sources of HPV information and formats of education and training to provide a comprehensive view of participants’ HPV-related education.

This tool was piloted and validated among a group of 11 staff educators (school staff from regions not included in the study sample) identified and contacted by the researchers through convenience sampling. The informed consent and questionnaire tools were administered via an online pilot questionnaire emailed for self-administration on a personal smart device. The piloted questionnaire included five sections: participant demographics, school structure, HPV awareness and education, HPV knowledge and HPV vaccination health beliefs and intentions. Written and oral feedback provided by the pilot participants allowed for minor adjustments in the simplification of wording of the questionnaire. It was identified within the pilot group that ‘Pap smear’ was the preferred terminology for cervical cytology, and this was adopted in the questionnaire.

Data were collected and managed using REDCap (Research Electronic Data Capture), a secure, web-based platform. Research Electronic Data Capture provides tools for building and managing online surveys and databases. An in-person study recruitment and data collection launching event was hosted for each of the participant groups, and an open Wi-Fi network was made available to participants to complete the survey during in-person study recruitment.

Follow-up invitations to participate in the study were distributed via email and the instant messaging platform, WhatsApp, during the 2-month data collection period. Invitations were distributed weekly to principals via email, and weekly invitation posters with an accessible link were distributed via the Health Promoting Schools of Mitchells Plain WhatsApp platform created and moderated by the school health nurse of Mitchells Plain, who coordinates the HPV vaccine campaign in the area; members of this instant messaging group consist exclusively of the local school health nurse and the SHRs recruited for this study. The overall survey response rate was 56.1% of the estimated 98 participants recruited. Of the 77 questionnaires submitted, 55 were completed correctly, yielding a completion rate of 71.4%. Twenty-two incomplete questionnaires were discarded to ensure that the analysis was based solely on complete responses.

### Data analysis

Numerical variables were summarised using medians, means and standard deviations, while categorical variables were reported as percentages and frequencies. *p*-values of less than or equal to 0.05 were regarded as statistically significant. Chi-square tests were applied to assess associations between categorical variables, while *t*-tests were used for numerical variables. These tests examined the association between demographic factors, participants knowledge, beliefs and health intentions and the association between receiving a cervical smear and participants knowledge scores. Group variances of categorical outcome variables were assessed for significant differences in group means across demographic factors, participants knowledge, beliefs and intention to recommend the HPV vaccine. Logistic regression was applied to categorical outcome variables of interest (‘Intention to recommend the HPV vaccine’ and ‘HPV health beliefs’) to explore for associations between outcome and predictor variables.

### Data management

Data from the questionnaires were captured using the REDCap web application. These data were exported in .CSV format and imported into Jamovi software (version 2.5 2024) for statistical analysis.

### Validity and reliability

Validated tools used in previous studies on educators regarding HPV and HPV vaccine knowledge, beliefs and attitudes were adapted to construct the data collection tool used in this study. To maintain the validity of the questionnaire and avoid loss of meaning during translation, the study material was administered in English, which is the second most dominant language within the study population. The questionnaire was standardised providing all participants with the same questions with clear instructions and guidance on what to expect. The full questionnaire administered to participants is appended as supplementary material (Appendix 1). Information collected and questions asked on the data collection tool addressed elements relevant to this study (i.e. HPV, HPV vaccination and cervical cancer knowledge, awareness, beliefs and attitudes). Before administration, the questionnaire was pilot-tested, and pilot test results were not included in this study. Convenience sampling was used, and all eligible principal or SHR staff who expressed willingness to participate were included in the study. Questions were constructed to be as clear as possible and free of specialised terminology, and leading questions were avoided. The questionnaire was anonymous, and participants were assured of confidentiality to minimise non-response bias. All data intended for collection in the study were collected and published.

### Ethical considerations

The study adhered to ethical standards by obtaining approval from the University of Cape Town Human Research Ethics Committee (International Review Board number: IRB00001938; HREC REF: 061/2024) and the Western Cape Education Department (reference number: 3A660222A0000008-20240417). Participation was anonymous and voluntary, ensuring that individuals could refuse or withdraw without negative consequences. Risks such as stigmatisation and loss of confidentiality were mitigated by using secure communication methods and anonymising participant data. Written informed consent was obtained via a detailed information sheet and consent forms. Electronically signed and dated consent was obtained from all participants who completed the survey using their personal smart devices. The survey was configured so that participants had to complete the consent fields before the questionnaire became available via the accessible link. Informed consent files were stored and anonymised on the REDCap system. The participants’ privacy was maintained by conducting the survey online, utilising number codes for anonymity and limiting data access to the research team. The study was low risk with minimal direct benefits, and no reimbursements were provided to participants.

## Results

### Demographics

Of the 55 complete responses collected, most participants were female (87.3%), and 40.0% of participants were aged 51 years–60 years. Despite being eligible for cervical smears, only 54.2% of female participants reported having ever undergone a cervical smear. A total of 21.8% of participants had either received the HPV vaccine themselves or reported that their child(ren) had received the vaccine. Most respondents were teachers (76.4%) (Table 1).

**TABLE 1:** Participant demographics (*N* = 55).

Frequency	*n*	%
**Sex**		
Female	48	87.3
Male	7	12.7
**Age (years)**		
21–30	11	20.0
31–40	12	21.8
41–50	9	16.4
51–60	22	40.0
≥ 61	1	1.8
**Undergone regular cervical smear exam**		
Yes	26	47.3
No	22	40.0
Cervical smear not applicable	7	12.7
**Received an HPV vaccine (participant or their child)**		
Yes	12	21.8
No	43	78.2
**Position**		
Teacher	42	76.4
Admin staff	5	9.1
Principal	8	14.5

HPV, human papillomavirus.

### School characteristics

Most of the participants were employed at primary schools (92.7%). Additionally, 7.3% of participants worked at combined schools (Grade 1–12). Among the respondents, 83.6% were employed at non-fee-paying schools, and 96.4% at WHO Health Promoting Schools (Table 2).

**TABLE 2:** Schools’ characteristics (*N* = 55).

Frequency	*n*	%
**School type**		
Combined (Grade 1–12)	4	7.3
Primary school	51	92.7
**Fee structure**		
Fee-paying	9	16.4
Non-fee paying	46	83.6
**Education system**		
Mainstream public school	51	92.7
Special education school	4	7.3
**WHO health promoting school**		
Health promoting	53	96.4
Non-health promoting	2	3.6
**School gender composition**		
Co-educational	55	100.0

WHO, World Health Organization.

### Knowledge scores

Overall, knowledge scores were low among all participant groups (median knowledge score = 7 out of 19 [interquartile range {IQR} = 4.0]) (Table 3). While 78.2% correctly identified the link between HPV infection and cervical cancer, just 18.2% recognised that infection from low-risk HPV strains is non-cancerous, and only 21.8% linked them to genital warts. The most poorly answered question required participants to correctly identify that cervical smears do not detect HPV directly but instead identify abnormal cervical cells that may reflect HPV-related changes (1.8%).

**TABLE 3:** Knowledge scores (*N* = 55).

Variables	Knowledge Score (out of 19)				
	*n*	Mean	s.d.	Min	Max
**Position**					
Teacher	42	6.98	2.97	1	14
Admin staff	5	9.40	3.05	6	13
Principal	8	6.25	4.68	0	13

**Total**	**55**	**7.09**	**3.29**	**0**	**14**

s.d., standard deviation; Min, minimum; Max, maximum.

Most participants (83.6%) correctly answered that women aged 30–60 are eligible for free cervical smear screening at a public health facility in South Africa. Participants who underwent regular cervical cytology scored higher (7.27) than those who did not (5.71). No statistically significant difference in test scores was found between those with or without a history of cervical cytology screening (*p* = 0.068). Notably, 38.2% of participants did not correctly identify that condoms can reduce the risk of HPV infection. The construction of the knowledge uncertainty variable is described in Appendix 2 and detailed scores per knowledge question are provided in Appendix 3, Table 1-A3.

### Knowledge uncertainty

The greatest uncertainty in knowledge was noted in questions relating to low-risk HPV infections, with 58.2% of participants choosing to respond ‘do not know’. Principals had the highest knowledge uncertainty scores (mean = 7.38, s.d. = 7.37) followed by the teachers’ group (mean = 6.93, s.d = 4.77), and administrative staff demonstrated the lowest uncertainty (mean = 4.80, s.d = 3.96) (Table 4). A statistically significant association (*p* = 0.002) was found between participant’s self-reported ability to guide learners about HPV vaccines and their own knowledge uncertainty. Similarly, there is a statistically significant association between self-perceived confidence in HPV-related knowledge among staff roles and their degree of knowledge uncertainty (*p* = 0.002). Participants with a high knowledge uncertainty were more likely to report not feeling equipped to guide their pupils about HPV (62.5%) compared to participants with moderate (54.8%) and low knowledge uncertainty (60.4%).

**TABLE 4:** Knowledge uncertainty (*N* = 55).

Variable	Knowledge uncertainty (out of 19)				
	*n*	Mean	s.d.	Min	Max
**Position**					
Teacher	42	6.93	4.77	0	17
Admin staff	5	4.80	3.96	0	11
Principal	8	7.38	7.37	0	19

**Total**	**55**	**6.80**	**5.09**	**0**	**19**

s.d., standard deviation; Min, minimum; Max, maximum.

Note: High knowledge uncertainty (10–19 ‘Do not know’). Moderate knowledge uncertainty (5–9 ‘Do not know’). Low knowledge uncertainty (0–4 ‘Do not know’).

Admin staff had the highest mean knowledge scores (9.40) and the lowest mean knowledge uncertainty score (4.80), in contrast with principals, who had the lowest mean knowledge scores (6.25) and the highest mean knowledge uncertainty score (7.38) (Table 3 and Table 4). Binomial regression analysis of knowledge and uncertainty as outcome variables did not derive statistically significant results.

### Health beliefs about and intention to recommend the human papillomavirus vaccine

Most participants (72.7%) strongly support the HPV vaccine for girls aged 9–10 years. Most disagreed that vaccine campaigns take up too much time (63.6%) and supported the continuation of school-based vaccination (89.1%). Ambivalence (‘neutral’ responses) was noted in 20.0% of participants regarding the vaccination of girls aged 9–10 years, with similar proportions among teachers (21.4%) and principals (20.0%), and lower among admin staff (12.5%). Ambivalence was also observed regarding sex education for girls aged 9–10 years (34.5% neutral; 43.6% agree). The summary of health beliefs results is described in Appendix 4, Table 1-A4.

Most participants reported they would recommend the HPV vaccine to students (85.5%), with 12.7% neutral and 1.8% who would not recommend the vaccine to their students (Table 5). Chi-squared tests of association and logistic regression did not derive statistically significant relationships among participants intention to recommend the HPV vaccine and campaign experience or demographic variables.

**TABLE 5:** Intention to recommend the human papillomavirus vaccine (*N* = 55).

Intention to recommend the HPV vaccine	*n*	%
Yes	47	85.5
I am not sure	7	12.7
No	1	1.8

HPV, human papillomavirus.

### Human papillomavirus vaccine campaign and training experience

Administrative staff had the longest experience in hosting HPV vaccination campaigns (mean = 7.0 years), followed by principals (mean = 3.63 years) and then teachers (mean = 3.12 years). Only 19.0% of teachers, 40.0% of support staff and 12.5% of principals had received formal training on HPV-related information. Most teachers (81%) reported no formal training on the subject (Table 6).

**TABLE 6:** Human papillomavirus campaign and training experience (*N* = 55).

Staff position	Vaccination campaigns during employment						Vaccination campaign involvement						Formal training			
	*n*	Mean	Median	s.d.	Min	Max	*n*	Mean	Median	s.d.	Min	Max	Yes		No	
													*n*	%	*n*	%
Teacher	42	5.2	5.0	3.2	0	12	42	3.1	3.0	2.9	0	11	8	19.0	34	81.0
Admin staff	5	7.6	9.0	3.4	3	11	5	7.0	7.0	3.2	3	11	2	40.0	3	60.0
Principal	8	5.0	4.0	3.0	2	11	8	3.6	2.5	3.6	0	11	1	12.5	7	87.5

s.d, standard deviation; Min, minimum; Max, maximum.

### Sources of information

**FIGURE 1:**
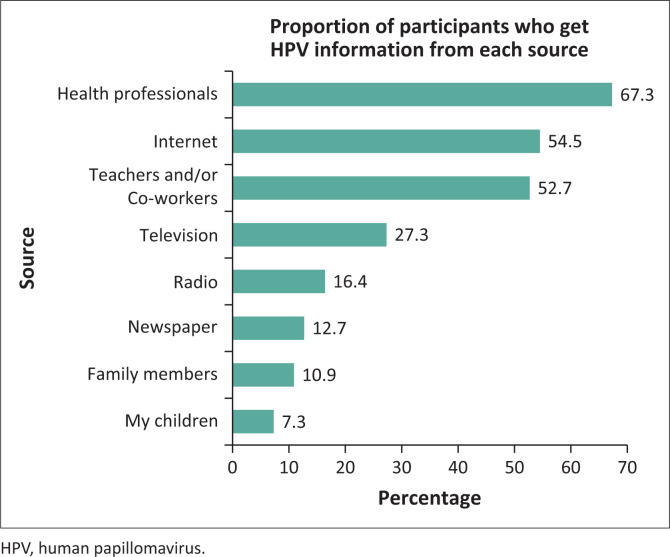
Proportion of participants who get human papillomavirus information from each source.

Participants identified multiple sources of information about the HPV vaccine, with the most common being health professionals (67.3%), the internet (54.5%) and teachers or co-workers (52.7%). Other sources included television, newspapers, radio, family members and children (Figure 1). Chi-squared test of association analysis and logistic regression analysis did not reveal statistically significant associations between HPV information sources variables and knowledge scores or intention to recommend HPV vaccination.

## Discussion

The school-based HPV vaccination campaign was introduced into South African public schools over 10 years ago. However, this study is the first of its kind to be administered locally to quantify the level of knowledge regarding HPV and the HPV vaccine. Concerningly, the results paint a picture of limited knowledge and notable uncertainty regarding HPV-related facts among the sampled SHRs and principals. They also demonstrate substantial ambivalence towards sexual and reproductive health education for children.

The lack of and uncertainty regarding HPV-related knowledge observed among participants is not unexpected in the context of an over-burdened health system. In Mitchells Plain, there are just two school nurses employed to serve 50 schools, and their responsibilities extend far beyond the HPV vaccination campaign. Quarterly information sessions with representative education staff must address a wide range of administrative and technical health-related issues. Consequently, the limited time available to address HPV-related matters is largely devoted to the logistics of the campaign rather than education. Currently, the school nurses have no way in which to assess health representatives’ HPV-related knowledge. Consequently, attendees may gain little additional knowledge, despite many of them attending these sessions every year.

In line with these observations, participants scored poorly in the knowledge section of the questionnaire (median score 7 out of 19; 36.8%). This mirrors findings based on similar questionnaires conducted in Kenya and Korea, where school staff scored 48% and 45.4% on average respectively (Kim 2012; Masika et al. 2015). Similarly, teachers in Malaysia also scored poorly on a questionnaire testing their HPV-related knowledge, although this study was conducted prior to the Malaysian government’s roll out of the country’s vaccination programme (Woo et al. 2012). In contrast to these studies, Greek educators participating in a study conducted by Xenaki et al. (2020) scored well on their knowledge tool. Participants in this study demonstrated limited knowledge regarding cervical cancer, as well as HPV infection, transmission and screening.

Most participants (78% – 83%) knew that the HPV vaccine reduces the risk of developing cervical cancer, but their knowledge was limited beyond knowing that cervical cancer can be fatal. This is similar to findings among participants by Masika et al. (2015). Of particular note is that only 61.8% of participants knew that condoms reduce the risk of HPV infection. Although relatively low, this proportion is considerably higher than that reported by Kim (2012), where only 16.6% answered correctly. This difference may reflect the longstanding health promotion campaigns in South Africa emphasising condoms as an effective strategy to prevent sexually transmitted infections (STI) in the context of the HIV epidemic. However, 74.5% of participants answered incorrectly when asked whether HPV is a STIs. While HPV is not exclusively transmitted sexually, the majority of oncogenic and genital HPV types are sexually acquired. This highlights an important gap in participants’ knowledge regarding STIs and their prevention.

Importantly, the lack of association between the number of campaigns participated in and knowledge scores achieved suggests that an assumption cannot be made that staff who have been involved in multiple campaigns are more knowledgeable. This reinforces the recommendation for targeted educational interventions along with a form of evaluation to assess the retention and application of concepts learnt. The observed association between participants’ self-perceived ability to guide learners about HPV-related queries and their own knowledge uncertainty suggests that participants are aware of their knowledge deficits. Only 9.1% of all participants reported that they have enough HPV-related information to be able to guide their pupils, with 80% expressing the desire to know more about HPV and the HPV vaccine. This is a finding replicated in multiple other studies, where despite lacking information, teachers are generally positive about the vaccine and would like to be better informed (Masika et al. 2015; Woo et al. 2012; Xenaki et al. 2020). Insufficient information has been frequently highlighted by parents as reasons for not allowing their children to be vaccinated (Ogilvie et al. 2010; Xenaki et al. 2020). This, in turn, emphasises the importance of teachers being equipped to provide accurate, up-to-date information to their pupils and their parents.

With respect to whether girls aged 9 years–10 years should receive sex education, this study found that a much lower proportion of participants were in favour of this than in the study by Masika et al. (2015) (45.2% vs. 79%, respectively). There was also a high proportion of participants (38.1%) who expressed ambivalence regarding this question, as opposed to just 10% found by Masika et al. (2015). Further qualitative research and community engagement would be valuable to understand the reasons behind this ambivalence. An encouraging finding in this study was that the HPV vaccination campaign is generally well supported among participants, although responses may have been influenced by social desirability bias. Most participants expressed support for the vaccination campaign (85.5%) and believed that school-based vaccination should continue (89.1%), although a small number of participants believed that the campaign takes up too much school time (7.3%). These findings are similar to those demonstrated in Kenya by Masika et al. (2015).

Despite these encouraging findings, the overall participation in this study was lower than anticipated. A possible explanation is self-selection bias, whereby individuals with more hesitant views towards HPV vaccination may have been less willing to participate. In addition, competing demands on school staff, limited perceived relevance of the topic and the personal nature of some questions may also have discouraged participation.

This study found that other teachers or co-workers were cited by just over half of participants as being sources of information regarding the HPV vaccine, alongside health professionals and the internet. The Kenyan study by Masika et al. (2015) similarly showed a high reliance on fellow teachers for information, with 30% of participants relying on colleagues. A study among teachers in the Mediterranean likewise found that educators frequently relied on visual and literacy-based media for HPV vaccine information (Keten et al. 2019). This highlights the need to strengthen the information provided to teachers, as doing so will not only better prepare them to address questions from students and parents, but also improve the accuracy of information disseminated among colleagues and through the media sources they commonly rely on.

### Limitations

The sample size was smaller than anticipated, and an estimated 25 schools (52.1%) of the 48 primary schools recruited participated in the study, which may limit the generalisability of the results. Low response rate in principals may have resulted in non-response bias and selection bias. Social desirability and recall bias may have influenced participants’ responses regarding their health beliefs and intention to recommend the HPV vaccine. The cross-sectional design of the study limits the ability to establish causality or determine temporal relationships.

## Conclusion

This study has shown conclusively that knowledge levels among SHRs and school principals in Mitchells Plain is limited. Consequently, it is recommended that increased emphasis is placed on providing accurate, up to date, evidence-based information to school staff about the vaccine to empower them to guide informed decision-making among fellow school staff, students and parents. Further interventional cohort research may help determine the effect of educational programmes on HPV-related knowledge, beliefs and vaccine advocacy.

## Appendix 1: Questionnaire

### Participant number ____

#### Section 1: For each question, choose the option that applies to you

1. ***What is your sex?***
  - Male
  - Female
2. ***Have you undergone regular pap testing?***
  - Yes
  - No
  - Pap testing does not apply to me
3. ***What is your age (please specify in years): ________________***
4. ***Have you or your children received an HPV vaccination?***
  - Yes
  - No
5. ***What is your current position at the school? Choose one of the following options:***
  - Teacher/Educator
  - Secretary/Administrative staff
  - Principal
6a. ***How many vaccination campaigns have occurred during your employment at the school? ________________________***
6b. ***How many vaccination campaigns have you been involved in during your employment at the school? ________________________***
7. ***How long have you been fulfilling the role of School Health Representative or principal? (years and/months i.e. 5 years and 2 months) ________________________***

#### Section 2: School Structure

The following questions pertain to the school that currently employs you; please choose the option that applies to you:

8. ***The school is a:***
  - Primary School
  - High School
  - Combined (Grade 1–12)
9. ***The school’s fee structure is:***
  - Fee-paying
  - Non-fee-paying
10. ***The school is a:***
  - Mainstream school
  - Special needs/ Special education school
11. ***The school is***
  - A health-promoting school
  - Not a health-promoting school
12. ***The school is a***
  - Co-ed school
  - Single sex school

#### Section 3: HPV Awareness and Education

13. ***For the following question, select every option that applies to you:I get my information about HPV, HPV vaccines and cervical cancer from:***
  - Television
  - Newspapers
  - Internet and/or social media
  - Doctors and/or nurses
  - Teachers and/or co-workers
  - Family members and/or relatives
  - My children.
14. ***Have you received any formal education and/or training regarding HPV vaccination, cervical cancer and the HPV vaccination programme in schools?***
  - Yes
  - NoIf you answered yes, in what format did this take place?
  - Structured course
  - Informal lectures and/or lessons
  - Pamphlets
  - Videos
  - Other: Please specify_______________________________________

#### Section 4: HPV Knowledge

The following questions are to give you the opportunity to demonstrate your knowledge of HPV. Please answer the following questions by indicating if the statement is true or false: HPV = Human papillomavirus, STIs = Sexually transmitted infections

15. ***Infection of the cervix with HPV is related to the development of cervical cancer.***
  - True (Correct = 1 point)
  - False
  - Do not know
16. ***Low-risk HPV infection does not cause cervical cancer.***
  - True (Correct = 1 point)
  - False
  - Do not know
17. ***HPV infection of the cervix is usually asymptomatic (does not show symptoms).***
  - True (Correct = 1 point)
  - False
  - Do not know
18. ***Only high-risk HPV infection causes warts around the genitalia.***
  - True
  - False (Correct = 1 point)
  - Do not know
19. ***Low-risk HPV infection causes dysplasia (abnormal changes of cells) of the cervical area.***
  - True
  - False (Correct = 1 point)
  - Do not know
20. ***HPV is a sexually transmitted infection (STI).***
  - True (Correct = 1 point)
  - False
  - Do not know
21. ***HPV can infect the oral cavity, respiratory tract and eyes.***
  - True (Correct = 1 point)
  - False
  - Do not know
22. ***Condoms decrease the risk of becoming infected with HPV.***
  - True (Correct = 1 point)
  - False
  - Do not know
23. ***HPV infection is related to sexual contact.***
  - True (Correct = 1 point)
  - False
  - Do not know
24. ***The incubation period (time between getting infected with a virus and developing symptoms) of HPV varies from several months to more than 1 year.***
  - True (Correct = 1 point)
  - False
  - Do not know
25. ***HPV can cause genital cancer in men.***
  - True
  - False (Correct = 1 point)
  - Do not know
26. ***If immunity is strong enough, HPV infection may gradually disappear.***
  - True (Correct = 1 point)
  - False
  - Do not know
27. ***HPV can be detected on a cervical cytology Pap exam.***
  - True
  - False (Correct = 1 point)
  - Do not know
28. ***Women aged 30–60 are eligible for a free pap smear at a public health facility in South Africa at least once every 10 years.***
  - True (Correct = 1 point)
  - False
  - Do not know
29. ***HPV infection can be treated with drugs and surgery.***
  - True
  - False (Correct = 1 point)
  - Do not know
30. ***Pregnant women with HPV infection can reduce the risk of their newborn getting HPV infection from vaginal delivery by having a Caesarean section instead.***
  - True (Correct = 1 point)
  - False
  - Do not know
31. ***Among those who develop cervical cancer, all of them had genital warts first.***
  - True
  - False (Correct = 1 point)
  - Do not know
32. ***HPV vaccination will reduce the risk of becoming infected with HPV.***
  - True (Correct = 1 point)
  - False
  - Do not know
33. ***HPV infection occurs mostly in women over the age of 25.***
  - True
  - False (Correct = 1 point)
  - Do not know

#### Section 5: Health Beliefs and Intentions

34. ***Would you recommend your students to get the HPV vaccination?***
  - Yes
  - No
  - I am not sure.
35. ***If your answer is NO, please indicate why? (Choose all that apply)***
  - I am against all vaccinations.
  - The vaccine is not safe.
  - The vaccine will make young girls start sexual activity early.
  - My religion does not allow vaccination.
  - The HPV vaccine is not necessary.
  - Other (specify): _________________________________________________

***For the each of the following statements, please choose one of the following: agree, disagree or neutral:***

**Table:**

	Agree	Disagree	Neutral (neither agree nor disagree)
**All 9–10-year-old girls should get the HPV vaccine.**			
**HPV infection is common in South Africa.**			
**I have enough information about HPV to guide my pupils.**			
**I would like to know more about the HPV vaccine.**			
**9– 10-year-old girls should get education about sex.**			
**Vaccine-related activities take up too much of the school’s teaching time.**			
**School-based vaccination of children should be continued.**			

**END OF QUESTIONNAIRE. Thank you for your time**

Link to informational video on HPV: https://youtu.be/qF7pBzU4D20?si=lYo1YmoO6Eia58FU. This 3-min video uses infographics to show how HPV may progress to cervical cancer, how the HPV vaccine works and how it was tested for safety and efficacy before it was approved. It was produced by the Danish Medicines Agency, which in partnership with World Health Organization Regional Office for Europe have now made it available for use in other countries.

## Appendix 2: Description of knowledge uncertainty variable construction

Knowledge uncertainty was stratified into high (10–19 ‘Do not know’), moderate (5–9 ‘Do not know’) and low (0–4 ‘Do not know’) uncertainty levels, using the 33rd and 66th percentiles as cut-offs, ensuring approximately equal-sized groups. A percentile-based approach was used instead of standard deviation–based cut-offs to avoid reliance on assumptions of normality. The total score, ranging from 0 to 19, reflected the sum of all correct responses.

## Appendix 3: Human papillomavirus knowledge scores per question

**TABLE 1-A3:** Participants’ knowledge of human papillomavirus infection and vaccination (*N* = 55).

Knowledge questions	Correct	
	*n*	%
Women aged 30–60 are eligible for a free pap smear at a public health facility in South Africa at least once every 10 years.	46	83.6
HPV vaccination will reduce the risk of becoming infected with HPV.	45	81.8
Infection of the cervix with HPV is related to the development of cervical cancer.	43	78.2
Condoms decrease the risk of becoming infected with HPV.	34	61.8
Among those who develop cervical cancer, all of them had genital warts first.	27	49.1
HPV infection is related to sexual contact.	26	47.3
HPV infection occurs mostly in women over the age of 25.	21	38.2
The incubation period (time between getting infected with a virus and developing symptoms) of HPV varies from several months to more than 1 year.	20	36.4
HPV infection of the cervix is usually asymptomatic (does not show symptoms).	19	34.5
HPV can cause genital cancer in men.	19	34.5
HPV can infect the oral cavity, respiratory tract and eyes.	18	32.7
HPV is a sexually transmitted infection (STI).	14	25.5
Pregnant women with HPV infection can reduce the risk of their newborn getting HPV infection from vaginal delivery by having a Caesarean section instead.	14	25.5
Only high-risk HPV infection causes warts around the genitalia.	12	21.8
If your immunity is strong enough, HPV infection may gradually disappear.	11	20.0
Low-risk HPV infection does not cause cervical cancer.	10	18.2
HPV infection can be treated with drugs and surgery.	6	10.9
Low-risk HPV infection causes dysplasia (abnormal changes of cells) of the cervical area.	5	9.1
HPV can be detected on a cervical cytology Pap exam.	1	1.8

*Source:* Adapted from Kim, H.W., 2012, ‘Knowledge about human papillomavirus (HPV), and health beliefs and intention to recommend HPV vaccination for girls and boys among Korean health teachers’, *Vaccine* 30(36), 5327–5334. https://doi.org/10.1016/j.vaccine.2012.06.040

## Appendix 4: Human papillomavirus health beliefs

**TABLE 1-A4:** Human papillomavirus vaccine health beliefs (*N* = 55).

Health belief statements	Agree		Disagree		Neutral	
	*n*	%	*n*	%	*n*	%
**All 9–10-year-old girls should get the HPV vaccine.**	**40**	**72.7**	**4**	**7.3**	**11**	**20.0**
Teacher	30	71.4	3	7.1	9	21.4
Admin staff	3	60.0	1	20.0	1	20.0
Principal	7	87.5	0	0.0	1	12.5
**HPV infection is common in South Africa.**	**35**	**63.6**	**2**	**3.6**	**18**	**32.7**
Teacher	26	61.9	2	4.8	14	33.3
Admin staff	5	100.0	0	0.0	0	0.0
Principal	4	50.0	0	0.0	4	50.0
**I would like to know more about the HPV vaccine.**	**44**	**80.0**	**2**	**3.6**	**9**	**16.4**
Teacher	34	81.0	2	4.8	6	14.3
Admin staff	4	80.0	0	0.0	1	20.0
Principal	6	75.0	0	0.0	2	25.0
**9–10-year-old girls should get education about sex.**	**24**	**43.6**	**12**	**21.8**	**19**	**34.5**
Teacher	19	45.2	7	16.7	16	38.1
Admin staff	2	40.0	2	40.0	1	20.0
Principal	3	37.5	3	37.5	2	25.0
**Vaccine-related activities take up too much of the school’s teaching time.**	**4**	**7.3**	**35**	**63.6**	**16**	**29.1**
Teacher	2	4.8	27	64.3	13	31.0
Admin staff	1	20.0	2	40.0	2	40.0
Principal	1	12.5	6	75.0	1	12.5
**School-based vaccination of children should be continued.**	**49**	**89.1**	**1**	**1.8**	**5**	**9.1**
Teacher	37	88.1	0	0.0	5	11.9
Admin staff	5	100.0	0	0.0	0	0.0
Principal	7	87.5	1	12.5	0	0.0
**I have enough information about HPV to guide my pupils.**	**5**	**9.1**	**32**	**58.2**	**18**	**32.7**
Teacher	4	9.5	24	57.1	14	33.3
Admin staff	1	20.0	2	40.0	2	40.0
Principal	0	0.0	6	75.0	2	25.0

*Source:* Adapted from Masika, M.M., Ogembo, J.G., Chabeda, S.V., Wamai, R.G. & Mugo, N., 2015, ‘Knowledge on HPV vaccine and cervical cancer facilitates vaccine acceptability among school teachers in Kitui County, Kenya’, *PLoS One* 10(8), e0135563. https://doi.org/10.1371/journal.pone.0135563

## References

[CIT0001] Amponsah-Dacosta, E., Blose, N., Kwinika, V.V. & Chepkurui, V., 2022, ‘Human papillomavirus vaccination in South Africa: Programmatic challenges and opportunities for integration with other adolescent health services’, *Frontiers in Public Health* 10, 799984. 10.3389/fpubh.2022.799984 35174123 PMC8841655

[CIT0002] Brabin, L., Stretch, R., Roberts, S.A., Elton, P., Baxter, D. & McCann, R., 2011, ‘The school nurse, the school and HPV vaccination: A qualitative study of factors affecting HPV vaccine uptake’, *Vaccine* 29(17), 3192–3196. 10.1016/j.vaccine.2011.02.038 21354481

[CIT0003] Bruni, L., Albero, G., Serrano, B., Mena, M., Collado, J.J., Gómez, D. et al., 2023, *Human papillomavirus and related diseases in South Africa*, ICO/IARC Information Centre on HPV and Cancer (HPV Information Centre), viewed 02 June 2023, from https://hpvcentre.net/statistics/reports/ZAF.pdf.

[CIT0004] Dorji, T., Nopsopon, T., Tamang, S.T. & Pongpirul, K., 2021, ‘Human papillomavirus vaccination uptake in low-and middle-income countries: A meta-analysis’, *EClinicalMedicine* 34, 100836. 10.1016/j.eclinm.2021.100836 33997733 PMC8102703

[CIT0005] Dubé, E., Gagnon, D., Clément, P., Bettinger, J.A., Comeau, J.L., Deeks, S. et al., 2019, ‘Challenges and opportunities of school-based HPV vaccination in Canada’, *Human Vaccines & Immunotherapeutics* 15(7–8), 1650–1655. 10.1080/21645515.2018.1564440 30633622 PMC6746476

[CIT0006] Etikan, I., Musa, S.A. & Alkassim, R.S., 2016, ‘Comparison of convenience sampling and purposive sampling’, *American Journal of Theoretical and Applied Statistics* 5(1), 1–4. 10.11648/j.ajtas.20160501.11

[CIT0007] Gallagher, K.E., Howard, N., Kabakama, S., Mounier-Jack, S., Burchett, H.E.D., LaMontagne, D.S. et al., 2017, ‘Human papillomavirus (HPV) vaccine coverage achievements in low and middle-income countries 2007–2016’, *Papillomavirus Research* 4, 72–78. 10.1016/j.pvr.2017.09.001 29179873 PMC5710977

[CIT0008] Hossain, M.B., Alam, M.Z., Islam, M.S., Sultan, S., Faysal, M.M., Rima, S. et al., 2021, ‘Health belief model, theory of planned behavior, or psychological antecedents: What predicts COVID-19 vaccine hesitancy better among the Bangladeshi adults?’ *Frontiers in Public Health* 16(9), 711066. 10.3389/fpubh.2021.711066 PMC8418098 34490193

[CIT0009] Jeudin, P., Liveright, E., Del Carmen, M.G. & Perkins, R.B., 2014, ‘Race, ethnicity, and income factors impacting human papillomavirus vaccination rates’, *Clinical Therapeutics* 36(1), 24–37. 10.1016/j.clinthera.2013.11.001 24417783

[CIT0010] Katz, I.T., Nkala, B., Dietrich, J., Wallace, M., Bekker, L.-G., Pollenz, K. et al., 2013, ‘A qualitative analysis of factors influencing HPV vaccine uptake in Soweto, South Africa among adolescents and their caregivers’, *PLoS One* 8(8), e72094. 10.1371/journal.pone.0072094 24023613 PMC3758285

[CIT0011] Keten, H.S., Ucer, H., Dalgaci, A.F., Isik, O., Ercan, Ö. & Guvenc, N., 2019, ‘Knowledge, attitude, and behavior of teachers regarding HPV (human papillomavirus) and vaccination’, *Journal of Cancer Education: The Official Journal of the American Association for Cancer Education* 36, 584–590. 10.1007/s13187-019-01668-2 31840211

[CIT0012] Khosa, L.A., Meyer, J.C., Motshwane, F.M.M., Dochez, C. & Burnett, R.J., 2022, ‘Vaccine hesitancy drives low human papillomavirus vaccination coverage in girls attending public schools in South Africa’, *Frontiers in Public Health* 10, 860809. 10.3389/fpubh.2022.860809 35685759 PMC9171038

[CIT0013] Kim, H.W., 2012, ‘Knowledge about human papillomavirus (HPV), and health beliefs and intention to recommend HPV vaccination for girls and boys among Korean health teachers’, *Vaccine* 30(36), 5327–5334. 10.1016/j.vaccine.2012.06.040 22749602

[CIT0014] Lenkokile, R., Hlongwane, P. & Clapper, V., 2019, ‘Implementation of the integrated school health policy in public primary schools in Region C, Gauteng province’, *African Journal of Public Affairs* 11(1), 196–211.

[CIT0015] Mann, C.J., 2003, ‘Observational research methods. Research design II: Cohort, cross sectional, and case-control studies’, *Emergency Medicine Journal* 20(1), 54–60. 10.1136/emj.20.1.54 12533370 PMC1726024

[CIT0016] Masika, M.M., Ogembo, J.G., Chabeda, S.V., Wamai, R.G. & Mugo, N., 2015, ‘Knowledge on HPV vaccine and cervical cancer facilitates vaccine acceptability among school teachers in Kitui County, Kenya’, *PLoS One* 10(8), e0135563. 10.1371/journal.pone.0135563 26266949 PMC4534439

[CIT0017] Newman, P.A., Logie, C.H., Lacombe-Duncan, A., Baiden, P., Tepjan, S., Rubincam, C. et al., 2018, ‘Parents’ uptake of human papillomavirus vaccines for their children: A systematic review and meta-analysis of observational studies’, *BMJ Open* 8(4), e019206. 10.1136/bmjopen-2017-019206 PMC5914890 29678965

[CIT0018] Ngcobo, N.J., Burnett, R.J., Cooper, S. & Wiysonge, C.S., 2018, ‘Human papillomavirus vaccination acceptance and hesitancy in South Africa: Research and policy agenda’, *South African Medical Journal* 109(1), 13. 10.7196/SAMJ.2018.v109i1.13723 30606297

[CIT0019] Ogilvie, G., Anderson, M., Marra, F., McNeil, S., Pielak, K., Dawar, M. et al., 2010, ‘A population-based evaluation of a publicly funded, school-based HPV vaccine program in British Columbia, Canada: Parental factors associated with HPV vaccine receipt’, *PLoS Medicine* 7(5), e1000270. 10.1371/journal.pmed.1000270 20454567 PMC2864299

[CIT0020] Olorunfemi, G., Ndlovu, N., Masukume, G., Chikandiwa, A., Pisa, P.T. & Singh, E., 2018, ’Temporal trends in the epidemiology of cervical cancer in South Africa (1994–2012)’, *International Journal of Cancer* 143(9), 2238–2249. 10.1002/ijc.31610 29786136 PMC6195436

[CIT0021] South African National Department of Health, 2017, *Cervical cancer prevention and control policy*, viewed 02 June 2023, from https://www.health.gov.za/wp-content/uploads/2021/07/cervical-cancer-policy.pdf.

[CIT0022] Statistics South Africa, 2011, *Census 2011: Mitchells plain*, viewed 30 December 2023, from https://www.statssa.gov.za/?page_id=4286&id=329.

[CIT0023] Statistics South Africa, 2023, *Cancer in South Africa (2008–2019)*, South African Government, Pretoria, viewed 02 June 2023, from https://www.statssa.gov.za/publications/03-08-00/03-08-002023.pdf.

[CIT0024] Western Cape Department of Health and Wellness, 2016, *Annual report 2015–2016*, Western Cape Government: Health, Cape Town, viewed 10 December 2023, from https://d7.westerncape.gov.za/sites/www.westerncape.gov.za/files/annual_report_2015_16.pdf.

[CIT0025] Western Cape Department of Health and Wellness, 2020, *COVID–19 population data: Circular H102 / 2020*, Western Cape Government: Health, Cape Town, viewed 09 December 2023, from https://d7.westerncape.gov.za/assets/departments/health/h_102_2020_covid-19_population_data.pdf

[CIT0026] Western Cape Department of Health and Wellness, 2023, *Annual report 2022–2023*, Western Cape Government: Health, Cape Town, viewed 10 December 2023, from https://provincialgovernment.co.za/department_annual/1427/2023-western-cape-health-and-wellness-annual-report.pdf.

[CIT0027] Whelan, N.W., Steenbeek, A., Martin-Misener, R., Scott, J., Smith, B. & D’Angelo-Scott, H., 2014, ‘Engaging parents and schools improves uptake of the human papillomavirus (HPV) vaccine: Examining the role of the public health nurse’, *Vaccine* 32(36), 4665–4671. 10.1016/j.vaccine.2014.06.026 24992714

[CIT0028] Woo, Y.L., Razali, S.M., Chong, K.R. & Omar, S.Z., 2012, ‘Does the success of a school-based HPV vaccine programme depend on teachers’ knowledge and religion? – A survey in a multicultural society’, *Asian Pacific Journal of Cancer Prevention* 13(9), 4651–4654. 10.7314/APJCP.2012.13.9.4651 23167396

[CIT0029] Xenaki, D., Plotas, P., Michail, G., Poulas, K. & Jelastopulu, E., 2020, ‘Knowledge, behaviours and attitudes for human papillomavirus (HPV) prevention among educators and health professionals in Greece’, *European Review of Medical and Pharmacological Sciences* 24(14), 7745–7752. 10.26355/eurrev_202007_22277 32744701

[CIT0030] Zhao, M., Wu, Q., Hao, Y., Hu, J., Gao, Y., Zhou, S. et al., 2021, ‘Global, regional, and national burden of cervical cancer for 195 countries and territories, 2007–2017: Findings from the global burden of disease study 2017’, *BMC Women’s Health* 21(1), 419. 10.1186/s12905-021-01571-3 34922503 PMC8684284

